# Lethal Hemorrhagic Disease and Clinical Illness Associated with Elephant Endotheliotropic Herpesvirus 1 Are Caused by Primary Infection: Implications for the Detection of Diagnostic Proteins

**DOI:** 10.1128/JVI.01528-19

**Published:** 2020-01-17

**Authors:** Angela Fuery, Taylor Pursell, Jie Tan, Rongsheng Peng, Peter D. Burbelo, Gary S. Hayward, Paul D. Ling

**Affiliations:** a Molecular Virology and Microbiology, Baylor College of Medicine, Houston, Texas, USA; b Dental Clinical Research Core, NIDCR, NIH, Bethesda, Maryland, USA; c Viral Oncology Program, Johns Hopkins School of Medicine, Baltimore, Maryland, USA; University of California, Irvine

**Keywords:** antibody, elephant, herpesvirus, immunoserology

## Abstract

Elephant endotheliotropic herpesvirus (EEHV) can cause lethal hemorrhagic disease in juvenile Asian elephants, both in captivity and in the wild. Most deaths associated with the virus are caused by two chimeric variants of EEHV1 (EEHV1A and EEHV1B), while two other EEHVs endemic within Asian elephants (EEHV4 and EEHV5) have been recognized but cause death less often. Whether lethal EEHV infections are due to primary infection or reactivation of latent virus remains unknown, and knowledge of the anti-EEHV antibody levels in young elephants is limited. To close these gaps, we sought to develop a serologic assay capable of distinguishing among infections with different EEHVs using a luciferase immunoprecipitation system (LIPS) for antibody profiling and a panel of conserved EEHV recombinant proteins and proteins unique to EEHV1. The results showed that elephants dying from EEHV1 hemorrhagic disease or ill from EEHV infection were seronegative for the EEHV species that caused the disease or illness, indicating that the events were associated with primary infection rather than reactivation of latent virus. We also demonstrated that waning of EEHV1-specific antibodies can occur in the first 2 years of life, when a threshold protective level of antibody may be needed to prevent severe EEHV1-related disease. Use of the LIPS assay to identify putative “diagnostic” proteins would be a valuable asset in determining the EEHV immune status of young elephants and responses to candidate EEHV vaccines in the future.

**IMPORTANCE** Whether clinical illness and deaths associated with elephant endotheliotropic herpesvirus (EEHV) infection result from primary infection or reactivation of latent virus is a longstanding question in the field. By applying a relatively new assay, the luciferase immunoprecipitation system (LIPS), combined with the genomic sequences of the viruses, we gained the insights and tools needed to resolve this issue. Our EEHV1-specific LIPS assay should be useful for assessing the vulnerability of elephant calves to infection with different EEHVs and evaluating antibody responses to anti-EEHV vaccines. A significant proportion of the Asian elephant population is under some form of human care. Hence, the ability to screen for EEHV immune status in elephant calves should have a major impact on the management of these animals worldwide.

## Abstract (precis)

Whether clinical illness and deaths associated with elephant endotheliotropic herpesvirus (EEHV) infection result from primary infection or reactivation of latent virus is a longstanding question in the field. By applying a relatively new assay, the luciferase immunoprecipitation system (LIPS), combined with the genomic sequences of the viruses, we gained the insights and tools needed to resolve this issue. Our EEHV1-specific LIPS assay should be useful for assessing the vulnerability of elephant calves to infection with different EEHVs and evaluating antibody responses to anti-EEHV vaccines. A significant proportion of the Asian elephant population is under some form of human care. Hence, the ability to screen for EEHV immune status in elephant calves should have a major impact on the management of these animals worldwide.

## INTRODUCTION

Elephant endotheliotropic herpesviruses (EEHV) can cause lethal hemorrhagic disease (EEHV-HD) in endangered Asian elephants, both in captivity and in the wild. Almost all deaths from EEHV infection in Asian elephants are caused by two chimeric variants of EEHV1—EEHV1A and EEHV1B (1, 2); both EEHV4 and EEHV5 are also endemic in Asian elephants but have only rarely been fatal (3, 4). EEHV2, -3, -6, and -7 are recognized as endemic in African elephants (1), but to date, hemorrhagic disease caused by these viruses has not been documented to the same extent as for EEHVs in Asian elephants. Recently, however, lethal (J. Proudfoot and M. Fayette, unpublished data) and nonlethal (5) cases of EEHV3 infection have been observed, raising increased concern over EEHV-HD in these elephants, as well. Among Asian elephants, juveniles between the ages of 2 and 8 years appear to be most vulnerable to EEHV-HD (1). The mechanisms contributing to this increased risk remain unknown but could involve a lack of preexisting anti-EEHV cellular (6) or humoral responses (or both). Cases of EEHV-HD generally do not occur before an elephant reaches 1 year of age, so it is reasonable to assume that some component of protection may come either from maternal antibodies obtained through the placenta or from breast milk before an elephant is weaned, although a previous study indicated that transplacental antibody transfer is more likely (7). If antibodies indeed confer protection from EEHV, or at least indicate protection from EEHV-HD, then it becomes important to measure these responses, not only to establish susceptibility to EEHV-HD or seropositivity, but to assess vaccine responses when vaccines for EEHV-HD are eventually produced.

A previous study developed a novel capture enzyme-linked immunosorbent assay (ELISA) for detection of IgG antibodies to EEHV glycoprotein B (gB) (8). The results showed that as many as 80% of elephants tested in North American and European zoos were EEHV seropositive. A follow-up study using the same assay determined a seroprevalence of 42.3% among captive Asian elephants in Thailand (9). Because gB is relatively well conserved, the possibility that serologic responses detected against the protein can distinguish among infections with the different EEHVs endemic within Asian elephant populations appears remote. We therefore sought to improve upon the first-generation assay by relying on EEHV antigens produced in mammalian cells rather than bacteria and increasing the dynamic range and number of EEHV antigens for interrogation. Antibody responses were assessed with the luciferase immunoprecipitation system (LIPS) assay (10). This assay has been used successfully in studies of human herpesviruses and other pathogens (11 – 16) and does not require species-specific detection reagents, making it an excellent candidate for assessing Asian elephant antibody responses.

To distinguish among responses to infection with different EEHV species, we studied proteins expressed by all EEHVs, as well as those unique to EEHV1A and -B. The primary goal was to determine which elephants were seropositive for EEHV1A, then EEHV1B, and ultimately other EEHVs. Our findings have allowed us to gain insight into the role of the antibody response in protecting against lethal EEHV-HD and how the disappearance of EEHV-specific antibodies obtained transplacentally could affect the susceptibility of young elephants to EEHV-HD after they reach 2 years of age. Finally, our results with the LIPS assay pave the way for wider use of the method in understanding the serology of EEHV and as a diagnostic tool for use at zoos and other institutions that house elephants at risk of contracting lethal EEHV-HD.

## RESULTS

### The LIPS assay is sensitive and specific for EEHV infection through detection of antibodies specific for U39, U28, and U14.

**FIG 1:**
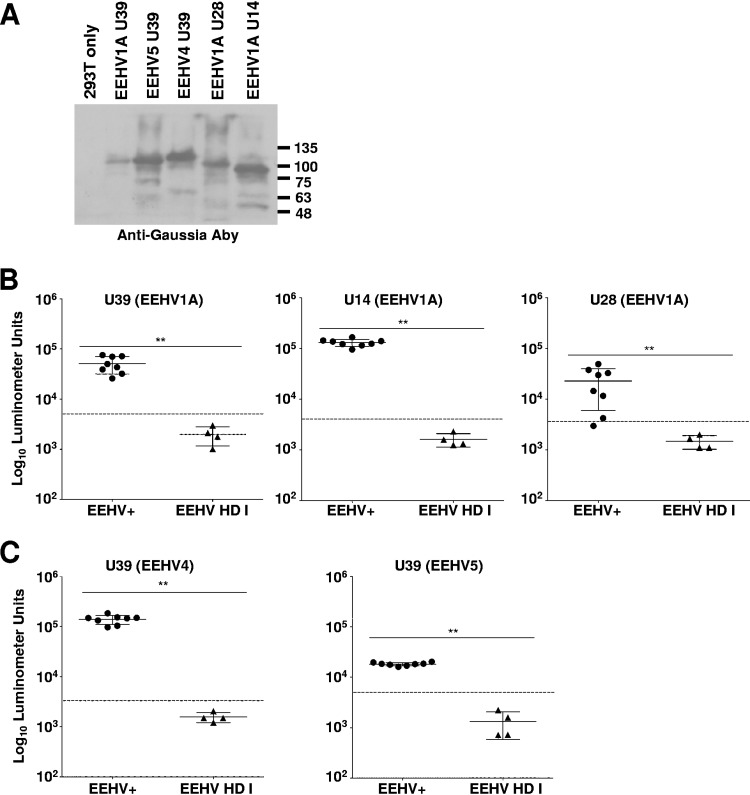
Detection of anti-EEHV antibodies by LIPS assays. (A) Immunoblot of EEHV-Gaussia luciferase fusion proteins detected with anti-Gaussia luciferase antibodies (Aby). Proteins are labeled at the top of the blot, with the sizes of protein markers (in kilodaltons) indicated on the right. (B and C) Detection of anti-EEHV1A U39, U14, and U28 antibodies (B) and anti-EEHV4 and 5 U39 antibodies (C) by LIPS assays. Each symbol represents samples from animals with evidence of prior EEHV infection(s) (EEHV+) and those that died from EEHV-HD (EEHV-HD group I). Antibody levels are plotted on the *y* axis using a log_10_ scale. Mean values ± SD for each cohort (EEHV+ or EEHV-HD group I) are shown, with each symbol representing the mean result for 1 elephant in at least 3 replicate experiments; the asterisks indicate a statistically significant difference (**, *P* < 0.005) between the EEHV+ and EEHV-HD I groups, as determined by the Mann-Whitney U test on log-transformed values. The dashed lines indicate the cutoff levels for determining the sensitivity and specificity for each viral antigen and are derived from the mean antibody titer of the uninfected samples plus 5 SD.

To establish the LIPS assay for use in Asian elephants, we assessed responses in two distinct cohorts: (i) adult elephants from a single herd with known exposure to one or more EEHVs (positive trunk washes or prior viremia), making them more likely to be chronically infected and to have an anti-EEHV antibody response (Table 1) (17 – 22), and (ii) elephants that died from EEHV-HD (Table 2). Of note, 7 of the 8 EEHV-HD cases investigated in this study were confirmed to have been associated with EEHV1A infection, while the remaining case was caused by EEHV1B (Table 2). We hypothesized that the EEHV-HD in the second cohort was due to a primary infection and therefore should be seronegative for general conserved EEHV proteins. Serum samples for testing in this cohort were obtained within a range of 1 day to 11 months before death and at an average of 6 years of age (Table 2). None of the elephants listed in Table 2 had detectable EEHV1 in their sera or blood by quantitative PCR (qPCR) in the weeks or months prior to their illnesses (data not shown). We initially chose 3 EEHV1A proteins—U39 (gB), U28 (ribonucleotide reductase subunit 1 [RRS1]), and U14 (tegument protein)—as targets to interrogate antibody responses representing known serological targets for other herpesviruses. These proteins are generally well conserved between EEHV1A and -1B, between EEHV1 and -5, and between EEHV1 and -4 and were likely to detect responses that were generated from infections with all known EEHVs (Table 3). Each of these potential antigens was expressed as a fusion protein with Gaussia luciferase (Gluc) for the LIPS assay, as described previously (10) (Fig. 1A). The LIPS assay was validated in Asian elephants by detecting a significant difference between the putative EEHV1A-seropositive and -seronegative cohorts for the conserved EEHV proteins U39 (*P* < 0.005), U14 (*P* < 0.005), and U28 (*P* < 0.005) (Fig. 1B). Only 4 elephants within the EEHV-HD cohort (HZI-10, -11, and -12 [Houston, TX, zoo {HZI}] and OKC-4 [Oklahoma City, OK, zoo {OKC}]) were compared in these initial experiments, because they appeared to be completely seronegative for the 3 EEHV proteins tested; they are referred to here as EEHV-HD group I. We did detect positive gB responses in 4 of the EEHV-HD cases (HZI-9, ABQ-1 [Albuquerque, NM, BioPark {ABQ}], FE-1, and FE-2 [Feld Entertainment {FE}]), but for the general purposes of establishing a reference EEHV-seronegative cohort, these animals (EEHV-HD group II) were excluded and are considered in more detail below. To further validate the EEHV-seronegative status of the 4 EEHV-HD group I animals, we checked for antibodies reactive with prototypes of the EEHV4 and -5 gB proteins and again found antibodies in the seropositive cohort but not in the seronegative cohort (EEHV4, *P* < 0.005, and EEHV5, *P* < 0.005) with use of the relevant U39 proteins (Fig. 1C).

**TABLE 1:** Summary of features of chronically EEHV-infected elephants evaluated in these studies

Elephant	Location	Sex,^*a*^ age (yr)	Origin (at birth)	Evidence of:^*b*^		
				EEHV1A	EEHV1B	EEHV4/5
HZI-1 (Thai)	Houston	M, 52	Wild	+	+	+
HZI-2 (Methai)	Houston	F, 49	Wild	+	+	+
HZI-3 (Shanti)	Houston	F, 27	Captive	+	+	+
HZI-4 (Tess)	Houston	F, 37	Wild	+	+	+
HZI-5 (Tucker)	Houston	M, 11	Captive	+	+	+
HZI-6 (Baylor)	Houston	M, 7	Captive	−	+	+
HZI-7 (Tupelo)	Houston	F, 7	Captive	+	+	+
HZI-8 (Duncan)	Houston	M, 4	Captive	−	+	+

a M, male; F, female.

b Evidence of prior viremia or shedding associated with the EEHV species. +, evidence; −, no evidence.

**TABLE 2:** Summary of features of 8 EEHV-associated lethal HD cases in these studies

Elephant	Location	Sex,^*a*^ age (yr)	Origin (at birth)	EEHV species^*b*^	Strain^*c*^	EEHV serostatus^*d*^
HZI-10 (Kiba)	Houston	M, 9	Captive	1B	NAP14	I
HZI-11 (Kimba)	Houston	F, 13	Captive	1A	NAP23	I
HZI-12 (Singgah)	Houston	M, 6	Captive	1A	NAP17	I
OKC-4 (Malee)	Oklahoma City	F, 4	Captive	1A	NAP73	I
HZI-9 (Beau Thai)	Houston	M, 4	Captive	1A	NAP06	II
ABQ-2 (Daizy)	Albuquerque	F, 5	Captive	1A	NAP72	II
FE-2	Feld	M, 2.5	Captive	1A	NAP75	II
FE-3	Feld	M, 4	Captive	1A	NAP80	II

a M, male; F, female.

b EEHV species associated with lethal EEHV-HD.

c North American Proboscivirus case number.

d EEHV-seronegative (group I) and EEHV seropositive (group II).

**TABLE 3:** EEHV protein relatedness across EEHVs

Protein^*a*^	Amino acid sequence identity (%) between EEHVs:			
	1A/1A	1A/1B	1A/5	1A/4
U39 (gB)	>99	87	80	64
U28 (RRS1)	100	100	85	68
U14 (TEG)	92	89–94	49	37

a TEG, tegument protein.

**FIG 2:**
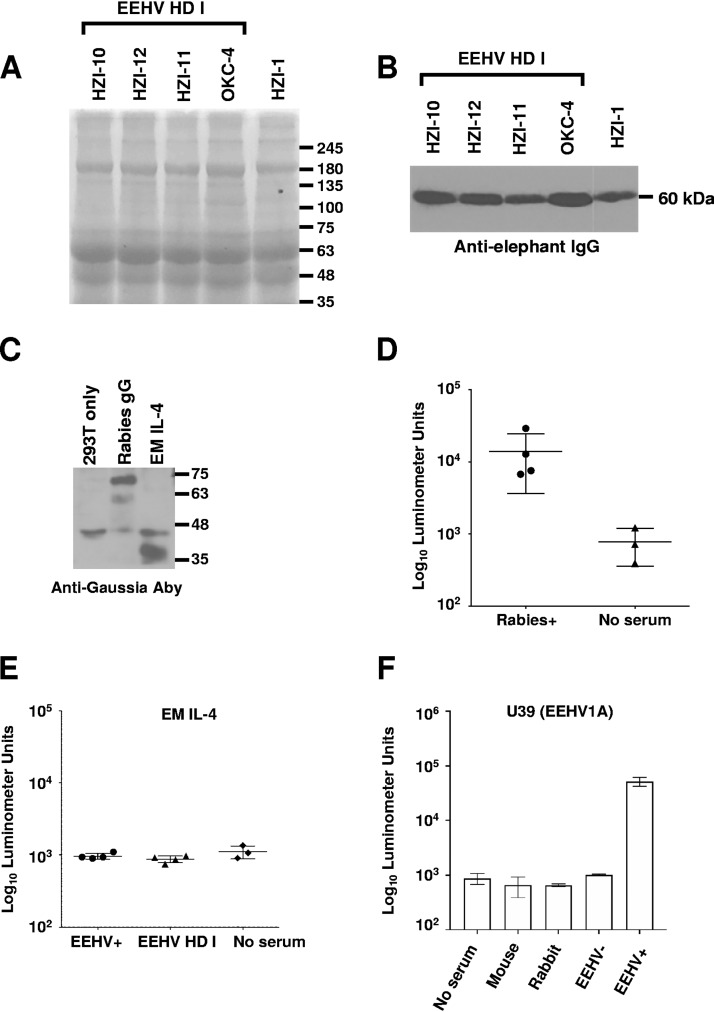
Detection of serum proteins and antibody activity in EEHV-seronegative sera. (A and B) Sera from EEHV-seronegative elephants (EEHV-HD group I) were characterized with Coomassie staining to identify intact proteins (values in kilodaltons) (A) and immunoblotting to identify immunoglobulin heavy chain (B). (C) Immunoblot of rabies virus glycoprotein G (gG) and elephant IL-4–Gaussia fusion proteins. (D) Antibodies specific for the rabies virus gG-Gaussia fusion were measured with LIPS in all EEHV-HD group I-seronegative elephants. (E) An elephant IL-4–Gaussia fusion protein was used to measure antibodies to an elephant protein in both seropositive and seronegative elephants, and the data were compared with those for no-serum controls. (F) The EEHV1A gB-Gaussia fusion protein was used to measure and compare antibodies between no-serum controls, mouse serum, rabbit serum, EEHV-seronegative serum (EEHV HD group I), and EEHV-positive serum. Antibody levels are plotted on a log_10_ scale. The mean ± SD for each cohort is shown, with each symbol representing the mean value for 1 elephant in at least 3 replicate experiments.

To confirm that the lack of detectable anti-EEHV antibodies in EEHV-HD group I elephants did not reflect the absence of any measurable antibodies, we analyzed the immunoglobulin contents of the serum samples. Coomassie staining and immunoblot analysis of sera from EEHV-seronegative elephants revealed intact immunoglobulin and heavy-chain immunoglobulin that were equivalent to findings in a recent sample from a healthy elephant (HZI-1) with measurable antibodies to EEHV proteins (Fig. 2A and B). We also detected a measurable antibody response to rabies virus glycoprotein G-Gaussia fusion protein by using LIPS to assess 4 presumptively EEHV-HD group I-seronegative elephants that had received rabies vaccine at least 2 years before their deaths (Fig. 2C and D). Moreover, an *Elephas maximus* interleukin 4 (IL-4)-Gaussia luciferase fusion protein in the LIPS assay showed no difference in antibody levels between the seropositive and seronegative elephant sera and yielded results similar to those for the no-serum controls (Fig. 2C and E). Finally, no-serum controls generated results similar to those obtained with sera from the seronegative (EEHV-HD I) cases and sera from healthy rabbits or mice and consistent with previous LIPS studies using no-serum controls with other antigens (Fig. 2F) (23, 24). Hence, to conserve valuable samples from the EEHV HD cohort, we elected to use no-serum controls as the basis for comparison instead of screening for IL-4-specific antibodies or testing the EEHV-seronegative cases.

**FIG 3:**
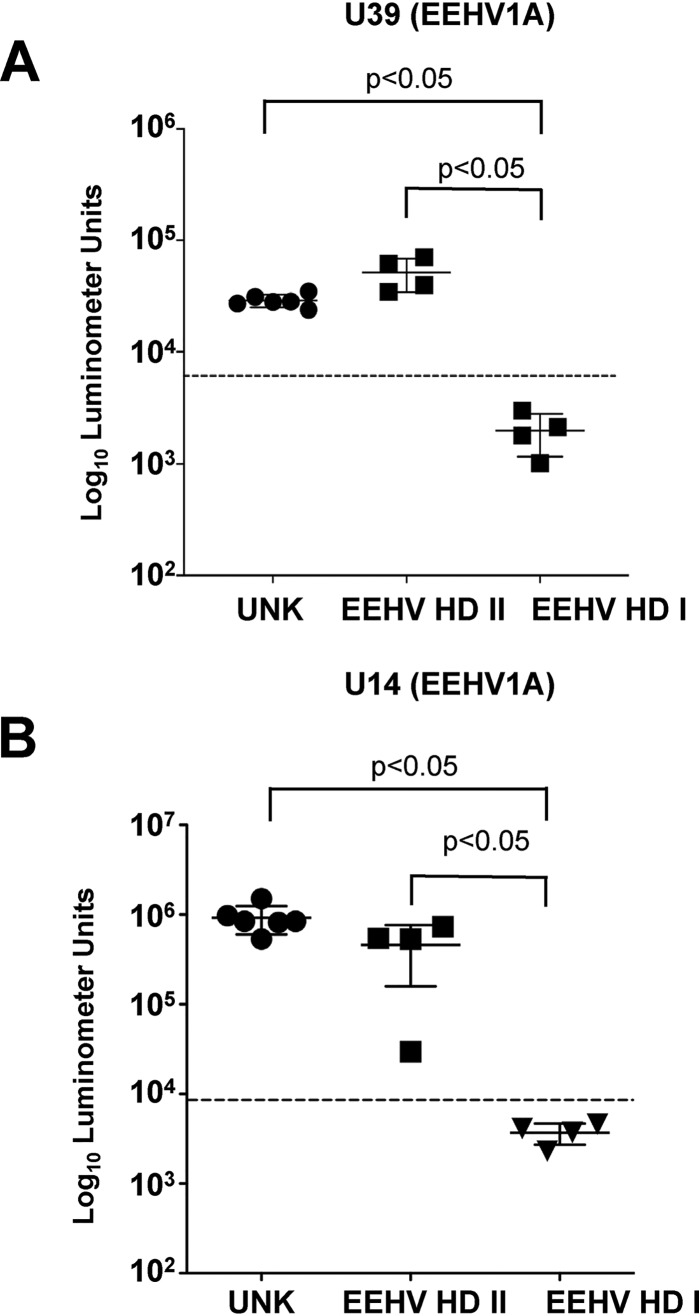
Detection of anti-EEHV antibodies in an uncharacterized cohort of adult elephants and in EEHV-HD-seronegative or -seropositive elephants. Shown is detection of anti-EEHV1A U39 antibodies (A) and anti-EEHV U14 antibodies (B) by LIPS assays. Each symbol represents samples from elephants living in herds with prior EEHV-HD infection (EEHV-HD group I and EEHV-HD group II) or with unknown status (UNK). Antibody levels are plotted on a log_10_ scale. The mean results ± SD for cohorts are shown, with each symbol representing the mean finding in 1 elephant in at least 3 replicate experiments. A *P* value of <0.05 indicates a statistically significant difference between the EEHV-HD I and EEHV-HD II groups and the uncharacterized adult cohort by the Mann-Whitney U test on log-transformed values. The dashed lines represent the cutoff levels for determining sensitivity and specificity for each viral antigen and are derived from the mean antibody titer of the uninfected samples plus 5 SD.

Having established assays sensitive for the detection of anti-EEHV antibodies, we used them to interrogate a cohort of adult elephants from 3 different herds (Table 4) and the 4 elephants in the EEHV-HD II group. In all instances, the elephants were immunoreactive to U39 and U14 at levels similar to the results for the HZI positive cohort (Fig. 3A and B). Thus, all of the adult elephants tested were immunoreactive to common conserved proteins encoded by the different EEHVs endemic within Asian elephant populations, whereas only a proportion of juveniles that succumbed to lethal infections showed evidence of infection with at least one EEHV type.

**TABLE 4:** Summary of features of elephants from different herds evaluated in these studies

Elephant	Location	Sex,^*a*^ age (yr)	Origin (at birth)	ORF-Q clade^*b*^
ABQ-1 (Alice)	Albuquerque	F, 45	Wild	D
ABQ-2 (Sampson)	Albuquerque	M, 21	Captive	D
OKC-1 (Asha)	Oklahoma City	F, 24	Captive	C
OKC-2 (Bamboo)	Oklahoma City	F, 53	Wild	C
OKC-3 (Chandra)	Oklahoma City	F, 23	Captive	C
FE-1	Feld	F, 22	Captive	C

a M, male; F, female.

b ORF-Q clade sequence was found in an EEHV-HD case(s) in the herd.

### Detection of anti-ORF-Q antibodies for the serologic diagnosis of EEHV1 infection.

**FIG 4:**
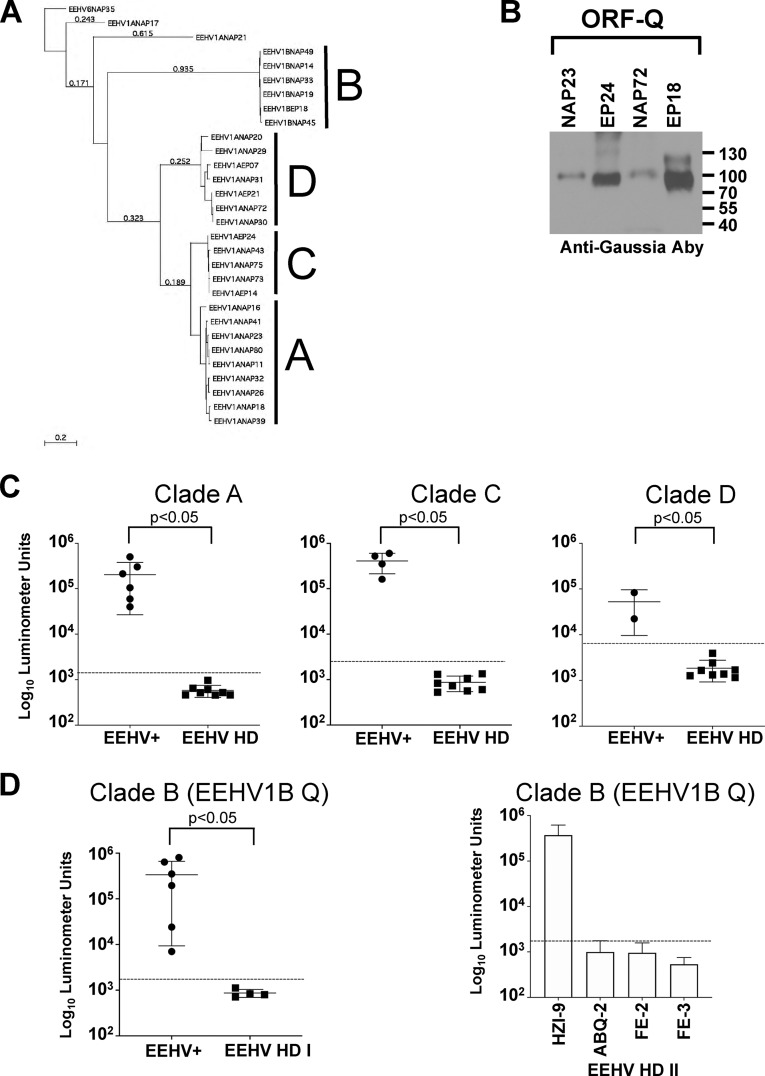
Antibody responses to ORF-Q protein identify prior exposure to EEHV1. (A) Phylogenetic tree of EEHV1 ORF-Q protein sequences derived from GenBank. Three major clades for EEHV1A strains are indicated (clades A, C, and D), while EEHV1B strains fall into a single clade (clade B). (B) Immunoblot analysis of ORF-Q–Gaussia fusion proteins representative of each clade. (C) Detection of anti-EEHV1A ORF-Q antibodies. The positive-control samples (EEHV+) were from herds where the circulating EEHV1A strains matching each clade, as listed in Tables 1 and 4, were compared with all of the EEHV-HD cohort (EEHV HD). (D) Detection of anti-EEHV1B ORF-Q antibodies. Positive controls from the HZI herd (Table 1) (EEHV+) were compared with the 4 EEHV-seronegative elephants from EEHV-HD group I. (Right) Anti-EEHV1B ORF-Q antibodies within EEHV-HD group II. Antibody levels are plotted on a log_10_ scale. Mean values ± SD for each cohort (EEHV+ or EEHV HD) are shown, with each symbol representing the mean of the results of at least 3 replicate experiments in a single elephant. A *P* value of <0.05 indicates a statistically significant difference between the EEHV+ and EEHV-HD groups as determined by the Mann-Whitney U test on log-transformed values. The dashed lines represent the cutoff levels for determining sensitivity and specificity for each viral antigen and are derived from the mean antibody titer of the uninfected samples plus 5 SD.

The cumulative evidence suggested that a significant proportion of lethal EEHV1A-HD cases (4 of 8 elephants tested [EEHV-HD group I]) occurred in elephants that appeared to be completely seronegative to any of the known EEHVs endemic within Asian elephant populations. However, 4 other EEHV1A-HD cases (EEHV-HD group II) did have antibodies to U39 and U14, indicating previous exposure to 1 or more of the 4 EEHVs (Fig. 3A and B). If these lethal cases were caused by primary infection, we predicted that the remaining 4 EEHV1A-HD cases would be EEHV1A seronegative. We searched, therefore, for proteins unique to EEHV1 to identify a biomarker capable of distinguishing EEHV1 infections from the other EEHVs. One protein, ORF-Q, is encoded by EEHV1 species only and has no known homology to any other proteins in GenBank. It has features of a glycoprotein but lacks any apparent transmembrane domain and functions in processes that are still undetermined. A potential limitation of using ORF-Q as a specific biomarker to detect EEHV1-specific antibody responses is its high variability among EEHV1 strains, particularly EEHV1A (Fig. 4A). To address this issue, we expressed 3 different ORF-Q–Gaussia fusion proteins that represent 3 major clades of known EEHV1A ORF-Q proteins (A, C, and D) (Fig. 4B), as well as the EEHV1A genotypes circulating within the 4 herds in which elephants had died from EEHV1A-HD (Tables 1 and 4).

To determine whether ORF-Q would detect antibody responses in infected elephants, we first tested the cohort of elephants selected from studies shown in Fig. 1, using Kimba EEHV1A ORF-Q (NAP23 [North American Proboscivirus {NAP}]), which is representative of clade A ORF-Q proteins and all 8 of the EEHV-HD sera. As shown in Fig. 4C, all 6 adult HZI elephants were highly immunoreactive against clade A ORF-Q, while all 8 EEHV-HD elephants from both groups I and II had no detectable responses. Similarly, elephants from herds with circulating clade C or clade D EEHV1A genotypes (represented by NAP72 and EP24 [European Proboscivirus {EP}], respectively) had high immunoreactivity to the corresponding ORF-Q proteins, whereas all 8 EEHV-HD elephants remained seronegative. To date, the intrastrain variability of the EEHV1B ORF-Q protein appears much lower than that observed for the EEHV1A strains; moreover, its homology with EEHV1A strains is only about 20%, suggesting that immunoreactivity to the protein might be able to distinguish between infections with the EEHV1A or EEHV1B variant of EEHV1. This hypothesis was tested first by comparing the immunoreactivity to EEHV1B ORF-Q in the Houston zoo cohort (Table 1), which is known to comprise multiple EEHV1B carriers, to that in the EEHV-seronegative EEHV-HD group I elephants. As expected, all the elephants from the EEHV1B-exposed herd showed high immunoreactivity to EEHV1B ORF-Q, while the EEHV-HD-seronegative animals (EEHV-HD group I) did not (Fig. 4D). EEHV-HD group II elephants were then tested, with one elephant (HZI-9) showing high immunoreactivity to EEHV1B ORF-Q protein (Fig. 4D). Thus, despite the presence of general anti-EEHV antibodies in some of the EEHV-HD elephants, a suggestive marker of infection by one or more of the EEHVs, all of the EEHV-HD cases were due to primary infection with EEHV1A or EEHV1B (i.e., HZI-10). Furthermore, the complete lack of immunoreactivity to any of the ORF-Q proteins in elephants ABQ-2, FE-2, and FE-3 suggests that in those animals, general reactivity to U39 and U14 (Fig. 3) reflected infection by EEHV4, EEHV5, or both.

**FIG 5:**
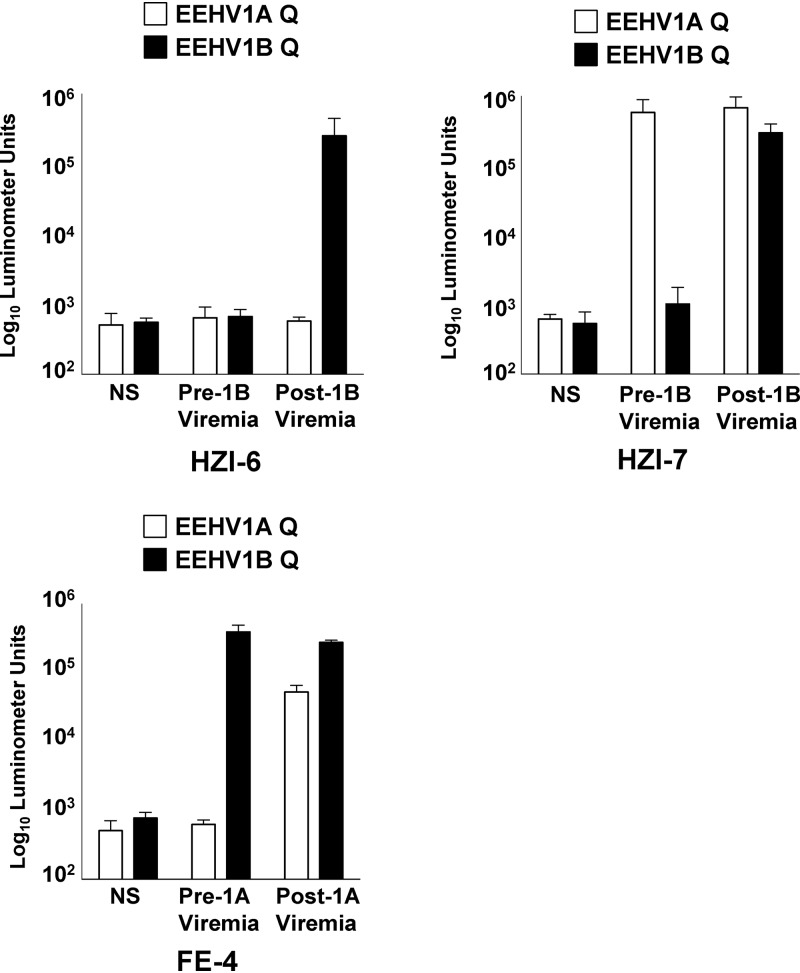
EEHV1A and EEHV1B ORF-Q antibody responses in serum samples pre- and post-EEHV1B and EEHV1A viremia. Antibodies specific for EEHV1A and EEHV1B variants of ORF-Q were measured in 3 elephants that survived acute EEHV1B (HZI-6 and HZI-7) and EEHV1A (FE-4) viremia. Antibody levels were measured by the LIPS assay. Serum samples were collected at least 1 month before EEHV1B viremia and at least 8 months after EEHV1A viremia. Each bar represents the mean finding in 1 elephant in at least 3 replicate experiments. The open bars represent immunoreactivity to EEHV1A ORF-Q, while the solid bars indicate reactivity to EEHV1A ORF-Q. Negative controls in which no serum (NS) was used are indicated.

To further establish that immunoreactivity to EEHV1A and EEHV1B ORF-Q proteins can distinguish between infections caused by these distinct EEHV species, we tested sera from 3 elephants before and after significant illness caused by either EEHV1B or EEHV1A. Although HZI-6 lacked antibodies to either EEHV1A or EEHV1B ORF-Q protein before the development of viremia and clinical illness due to EEHV1B (19), a distinct response to EEHV1B ORF-Q was seen after viremia in the absence of any response to EEHV1A ORF-Q (Fig. 5). Elephant HZI-7 already had antibodies specific for EEHV1A before EEHV1B viremia, but the EEHV1B ORF-Q response in the animal increased sharply after viremia without any substantial change in the EEHV1A response (Fig. 5). Finally, elephant FE-4 had preexisting immunoreactivity to EEHV1B ORF-Q, but not EEHV1A ORF-Q; however, after viremia and clinical illness due to EEHV1A (Dennis Schmitt, personal communication), there was a robust response to EEHV1A ORF-Q. Thus, in all 3 animals, specific seroconversion of the EEHV type to the virus causing viremia and illness occurred. In addition to the 8 lethal cases, these results suggest that nonlethal clinical illness can also result from primary EEHV1 infection.

### Decline of maternal anti-EEHV1 antibody titers in juvenile elephants.

**FIG 6:**
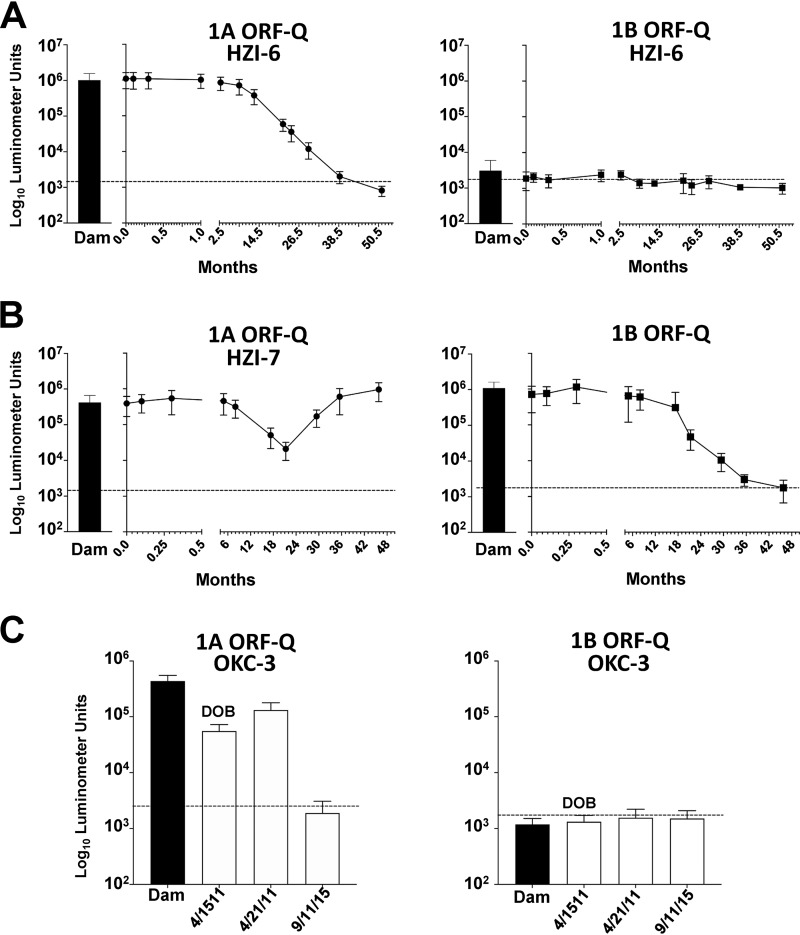
Longitudinal analysis of anti-EEHV1 maternal antibody levels over time in elephant calves. (A and B) Anti-EEHV1A and EEHV1B antibodies, as determined by immunoreactivity to EEHV1A ORF-Q or EEHV1B ORF-Q, from birth until just over 4 years in elephants HZI-6 (A) and HZI-7 (B). The solid bar in each graph represents the antibody levels detected in the dam on the day of parturition. (C) Comparative anti-EEHV1A and EEHV1B antibodies for OKC-4 and its dam at birth, day 7, and 14 days before EEHV-HD caused by EEHV1A. LIPS assays and cutoff points are described in the legend to Fig. 4.

A previous study determined that the majority of maternal-fetal antibody transfer is transplacental in elephants (7), although no specific test was available at the time to detect levels of anti-EEHV antibodies. Having established immunoreactivity to EEHV1 ORF-Q protein as a biomarker for prior EEHV1 infection, we asked whether elephant calves received anti-EEHV1 antibodies from their dams and how long these titers were sustained; elephants HZI-6 (Baylor), HZI-7 (Tupelo), and OKC-4 (Malee), which were surveyed in the previous study (7), were used to address these questions. We found that HZI-6 had anti-EEHV1A ORF-Q antibody levels at birth that equaled or slightly exceeded those found in the dam, which decreased to undetectable levels by approximately 36 months (Fig. 6A). In contrast, the dam lacked detectable antibodies to EEHV1B ORF-Q, a finding that corresponded to results in the calf (Fig. 6A). Notably, the calf did seroconvert to EEHV1B some months later, after viremia and illness associated with EEHV1B infection (Fig. 5). Elephant HZI-7 had antibody levels to both EEHV1A and EEHV1B ORF-Q at birth that were similar in magnitude to those of the dam (Fig. 6B). As anti-EEHV1A antibodies were declining with kinetics similar to those seen in elephant HZI-6, the animal experienced a dramatic spike in anti-EEHV1A ORF-Q antibodies at approximately 24 months, which has been sustained (data not shown). HZI-7 was being routinely monitored for EEHV1 viremia when it apparently seroconverted, but neither viremia nor clinical illness was observed (data not shown). In contrast to HZI-6, elephant HZI-7 received anti-EEHV1B antibodies, which declined to undetectable levels by approximately 36 months (Fig. 6B). Several months later, similar to elephant HZI-6, HZI-7 became ill due to EEHV1B infection, at which time there was seroconversion to EEHV1B (Fig. 5). Elephant OKC-4 had anti-EEHV1A antibody levels similar in magnitude to findings in the dam but lacked detectable anti-EEHV1B antibodies, matching the serologic profile of the dam (Fig. 6C). By 4 years of age, the elephant no longer had detectable anti-EEHV1A antibodies and ultimately succumbed to a lethal infection by EEHV1A (Table 1). These results indicate that elephant calves receive antibodies to EEHV transplacentally and that titers decline to undetectable levels by 36 months. The absence of detectable anti-EEHV1 antibodies correlated with development of clinical disease from primary infection with EEHV1.

## DISCUSSION

Whether EEHV-associated clinical illness or lethal hemorrhagic disease is caused by primary infection or reactivation of latent virus has remained unknown since the virus was discovered almost 2 decades ago. Clarification of this issue will provide important guidance for the development of improved treatments and generation of a vaccine. The results described here address the basis of lethal hemorrhagic disease or clinical illness associated with EEHV1A or EEHV1B in young elephants. Sensitive and specific serologic assays devised to detect broadly immunoreactive proteins for all of the EEHVs endemic within Asian elephant populations (Fig. 1 and 2), as well as assays specific for detection of antibodies against a protein uniquely encoded by EEHV1 species (Fig. 3 and 5), provide critical evidence that primary infection with the EEHV1A or EEHV1B variant of EEHV underlies these diseases. We also confirmed that transplacental transfer of anti-EEHV antibodies occurs and that these antibodies can wane over time to undetectable levels (Fig. 6), leading to a state of vulnerability to primary infections that can cause illness or death. Finally, the relatively simple LIPS test for ORF-Q antibodies appears to be a useful tool for assessing the immune status of young elephants at risk to develop severe EEHV infection.

The EEHV-specific LIPS assay has a number of features that support its use to interrogate the EEHV infection status of Asian elephants. First, the establishment of cutoff values that clearly identify positive and negative animals obviates the need to cope with the broad range of findings for some of the EEHV antigens tested (e.g., U14 and ORF-Q). Second, the production of antigens in mammalian cells most likely results in authentic posttranslational modifications compared to proteins made in prokaryotic systems; this feature may be particularly important for some of the viral glycoproteins tested. Third, because only small amounts of both serum and target fusion proteins are needed in the LIPS assay, it can provide detailed immunoprofiling with even the most limited serum samples. Finally, the production of multiple antigens for interrogation is very rapid, allowing ready incorporation of new antigens for more comprehensive immunoprofiling. In particular, we identified at least one antigen, ORF-Q, that is both unique to EEHV1 strains and highly immunogenic in elephants. Incorporation of this antigen into the LIPS assay provided a means to distinguish between infection with EEHV1 and infection with the other EEHV strains (i.e., EEHV4 and EEHV5). These findings were critical in addressing the origins of previous cases of EEHV infection, whether they were due to primary infection or reactivation.

Previous studies relied on an EEHV gB ELISA to show that 80% of elephants in North America and Europe and 42% within a large group of elephants tested in Thailand were seropositive for EEHV infection (8, 9). In our study, in contrast, 100% of healthy adult elephants had evidence of prior EEHV infection. One likely reason for the discrepancy could be that the LIPS fluid phase immunoassays we devised here have increased sensitivity and specificity compared with the first-generation solid-phase assay. In addition, the ability to profile multiple EEHV proteins, broadening the pool of potentially reactive antigens, allows the detection of heterogeneous immune responses by individual elephants within a population.

One limitation of our study is the small number of adult and juvenile animals tested. Although the serologic assays showed 100% sensitivity and specificity, we anticipate that with surveillance of larger numbers of animals, the range of antibody responses in elephants with evidence of chronic infection will likely increase. Whether this will alter the cutoff values in our current assays remains to be determined. Additional juvenile animals will need to be surveyed to determine whether the current time range for disappearance of maternally transferred antibodies (about 36 months) is broadly representative of most elephants.

One intriguing observation was the apparent EEHV1A seroconversion of elephant HZI-7 just before 24 months of age. While the elephant’s apparent EEHV1A-specific titers (assuming they were representative of titers to other EEHV1 proteins) had declined by almost 10-fold, they still remained more than 10-fold above the cutoff value. Importantly, EEHV viremia was not detected by routine weekly qPCR surveillance. It is tempting to speculate that anti-EEHV maternal antibodies may have played a protective role in limiting this case to a benign primary infection with EEHV1A. It should be emphasized that the anti-ORF-Q antibodies in elephant HZI-7 have remained at levels similar to the initial seroconversion levels. However, it seems fairly clear that once antibodies to EEHV1 wane to undetectable levels, some elephants appear to be susceptible to primary infections with the virus, leading to clinical illness and possible lethality.

While a considerable number of *ORF-Q* gene sequences from EEHV strains circulating in North American elephant herds (and to a more limited degree in European herds) have been identified, it remains unclear whether the four ORF-Q proteins representative of the major clades will be sufficient to detect responses to EEHV infections throughout the world. Although we have assumed that all elephants with earlier EEHV1A or EEHV1B infection produce antibodies to ORF-Q, additional EEHV1-specific biomarkers, as well as markers to specifically identify EEHV4 and/or EEHV5 infections, would likely be useful in avoiding misdiagnosis of elephants as seronegative or naive to EEHV1 infection.

Overall, our findings suggest that LIPS serologic assays are highly useful for identifying potentially EEHV-seronegative elephants or those that have not yet experienced infection with either EEHV1A or EEHV1B, which cause the majority of lethal EEHV-HD cases, and could be an important tool in elephant management. Examples include assessing the risk of transferring elephants between facilities and whether to initiate aggressive treatment for potential EEHV disease, which can involve a number of disruptive activities for both zoo personnel and the elephant herd. Finally, the assay should be of considerable value in assessing humeral immune responses to candidate anti-EEHV vaccines in future studies.

## MATERIALS AND METHODS

### Study sera.

Sera were obtained from 8 living Asian elephants at the Houston zoo that were latently infected with various EEHVs (seropositive), as well as 8 elephants that had died from HD caused by EEHV1 infection (seronegative). Additional sera were obtained from elephants at the Albuquerque BioPark, Oklahoma City zoo, and Feld Entertainment.

### Preparation of Ruc antigen fusion constructs.

Codon-optimized sequences were synthesized (GenScript) for predicted EEHV proteins and cloned in frame with the Gluc gene in the mammalian expression vector pGAUS3 (25, 26). EEHV1A U39, U14, U28, and ORF-Q (clade A) proteins were derived from EEHV1A Kimba (27) and the EEHV4 and EEHV5 U39 proteins from Vijay (4) and Baylor (28) genome sequences, respectively. ORF-Q proteins representative of clades B, C, and D were derived from sequences AGE09971.1, AGE10079.1, and APG41545.1, respectively. The predicted molecular weights of the Gaussia fusion proteins are listed in Table 5. Expression plasmids were transfected into 293T cells, and after 48 h, the supernatants and cell extracts were harvested for luminescence determination on a Glomax 20/20 luminometer (Promega). The extracts were stored at –80°C until they were required for LIPS analysis.

**TABLE 5:** Predicted molecular weights of Gaussia fusion proteins used in the LIPS assay

Protein	Gaussia fusion protein mol wt (kDa)
EEHV1A gB^*a*^	103
EEHV4 gB^*a*^	105
EEHV5 gB^*a*^	102
EEHV1A U14	85
EEHV1A U28	111
EEHV1A Kimba ORF-Q	56
EEHV1A Ramen ORF-Q	59
EEHV1A Daizy ORF-Q	60
EEHV1B Emelia ORF-Q	54
Rabies virus gG^*a*^	71
*E. maximus* IL-4	35

a Transmembrane and intracytoplasmic domains were deleted.

### LIPS analysis.

LIPS analysis was performed as previously described (29). Briefly, serum samples were initially diluted 1:10 in buffer A (50 mM Tris, pH 7.5, 100 mM NaCl, 5 mM MgCl_2,_ 1% Triton X-100) for storage for up to a month at 4°C. For use in the assay, samples were diluted 1:5 in the same buffer. Luminescence units (LU) of stored Gluc extracts were determined on the day of the assay, and a master mixture of each Gluc extract containing 1 × 10^7^ LU per 50 μl was made. Fifty microliters of Gluc extract master mixture was added to 50 μl of diluted serum (in duplicate) and incubated on a shaker at room temperature (RT) for 1 h. After 1 h, the Gluc extract and diluted serum were added to 5 μl of a 30% protein A-G bead suspension (diluted in phosphate-buffered saline [PBS]). Samples were incubated with the beads for 1 h at RT. The beads were then washed 3 times with buffer A, followed by 2 times with PBS. Fifty microliters of coelenterazine assay substrate was then added to the bead pellet, the tubes were vortexed, and LU values were immediately determined on a Glomax 20/20 luminometer (Promega) with a 5-s read time.

### Immunoblot assays.

Serum samples were mixed with 2× sample loading buffer, and proteins were resolved by SDS-PAGE as described previously (30). Some of the gels were analyzed by staining with PageBlue protein-staining solution according to the manufacturer’s recommendations (ThermoFisher). Proteins from a duplicate gel were transferred to nitrocellulose, and immunoblotting was performed with an anti-elephant IgG antibody as described previously (30). For analyses of EEHV Gaussia fusion proteins, supernatants or extracts from cell-associated Gluc fusions were mixed with 2× sample loading buffer and resolved by SDS-PAGE, and immunoblotting was carried out as described previously (30). A commercially available rabbit polyclonal anti-Gaussia antibody (New England Biolabs) was used to detect the EEHV-Gluc fusion proteins.

### Phylogenetic analysis of EEHV1 ORF Q proteins.

A linear-distance-based Bayesian phylogenetic tree comparison of the EEHV1 ORF Q proteins was generated with MacVector 12 as described previously (31). Accession numbers for each of the ORF Q protein sequences associated with the North American Proboscivirus (NAP) or European Proboscivirus (EP) cases used in this analysis are available in GenBank.

### Statistical analysis.

Statistical analysis was done with GraphPad (San Diego) Prism. In general, LIPS assays were performed in duplicate, with at least 3 independent experiments for each sample. Results for different groups are presented as geometric means ± the standard deviation (SD); in comparisons of group means with Mann-Whitney U tests, significance was set at a *P* value of ≤0.05. The cutoff level for determining sensitivity and specificity for each viral antigen was derived from the mean antibody titer of the uninfected samples plus 5 SD.
